# Thymine-Modified Nanocarrier for Doxorubicin Delivery in Glioblastoma Cells

**DOI:** 10.3390/molecules28020551

**Published:** 2023-01-05

**Authors:** Albina Y. Ziganshina, Elina E. Mansurova, Alexandra D. Voloshina, Anna P. Lyubina, Syumbelya K. Amerhanova, Marina M. Shulaeva, Irek R. Nizameev, Marsil K. Kadirov, Leysan R. Bakhtiozina, Vyacheslav E. Semenov, Igor S. Antipin

**Affiliations:** 1 Arbuzov Institute of Organic and Physical Chemistry, FRC Kazan Scientific Center, Russian Academy of Sciences, Arbuzov Str. 8, 420088 Kazan, Russia; 2 Alexander Butlerov Institute of Chemistry, Kazan Federal University, Lobachevsky Str. 1/29, 420008 Kazan, Russia; 3 Department of Nanotechnologies in Electronics, Kazan National Research Technical University Named after A. N. Tupolev—KAI, 10, K. Marx Str., 420111 Kazan, Russia

**Keywords:** glioblastoma, doxorubicin, nanocarrier, uracil, thymine, diallyl disulfide, drug delivery

## Abstract

Brain tumor glioblastoma is one of the worst types of cancer. The blood–brain barrier prevents drugs from reaching brain cells and shields glioblastoma from treatment. The creation of nanocarriers to improve drug delivery and internalization effectiveness may be the solution to this issue. In this paper, we report on a new nanocarrier that was developed to deliver the anticancer drug doxorubicin to glioblastoma cells. The nanocarrier was obtained by nanoemulsion polymerization of diallyl disulfide with 1-allylthymine. Diallyl disulfide is a redox-sensitive molecule involved in redox cell activities, and thymine is a uracil derivative and one of the well-known bioactive compounds that can enhance the pharmacological activity of doxorubicin. Doxorubicin was successfully introduced into the nanocarrier with a load capacity of about 4.6%. Biological studies showed that the doxorubicin nanocarrier composition is far more cytotoxic to glioblastoma cells (T98G) than it is to cancer cells (M-HeLa) and healthy cells (Chang liver). The nanocarrier improves the penetration of doxorubicin into T98G cells and accelerates the cells’ demise, as is evident from flow cytometry and fluorescence microscopy data. The obtained nanocarrier, in our opinion, is a promising candidate for further research in glioblastoma therapy.

## 1. Introduction

Glioblastoma is a malignant tumor with the highest mortality and low efficacy of anticancer therapy [1,2]. Glioblastoma has an infiltrative nature, so it is impossible to remove the entire tumor by surgical intervention. Known anticancer drugs demonstrate low efficiency and selectivity due to the low permeability of the blood–brain barrier (BBB) [3,4,5,6]. The development of nanocarriers for delivering drugs to the tumor is a promising and fast-evolving strategy [7]. Nanocarriers are necessary for boosting drugs’ bioavailability and for helping them get past physiological barriers. Additionally, drugs that are now not used to treat glioblastoma because of their high toxicity to healthy brain cells could be applied with nanocarriers that selectively target cancer cells [8]. One of these drugs is doxorubicin (DOX), an antitumor antibiotic that causes cell death by damaging DNA and preventing its replication [9,10]. DOX is a very effective medicine, but it poorly penetrates cancer cells and circulates throughout the body via blood flow, harming all crucial organs [11]. For this reason, in recent years, many studies have focused on the creation of DOX nanocarriers. To obtain them, micelles, liposomes, and dendrimers were used, the surfaces of which were modified with fragments facilitating passage through the BBB. These are proteins, sugars, acids, alcohols, and polyglycols [12,13,14,15,16,17,18,19,20,21,22]. The presented nanocarriers indeed improved the passage of DOX through the BBB, but unfortunately, most of them did not increase the effectiveness of therapy and did not demonstrate the benefits of their use [23,24]. Along with the passage of the BBB, there remain the problems of uncontrolled release of DOX, the low stability and accumulation of nanocarriers in nontarget areas, and their toxic effects on the body. For this reason, it is still necessary to look for novel solutions to the issue of developing a DOX nanocarrier. Nanoemulsions, which are stable liquid-in-liquid dispersions with a droplet size of roughly 100 nm, are one of the prospective nanocarriers [25]. They have been extensively developed in recent years for the binding and delivery of substrates, particularly drugs. Nanoemulsions have a stronger bond to the substrate than traditional carriers, and they require stimulation to release the substrate [26].

**Scheme 1:**
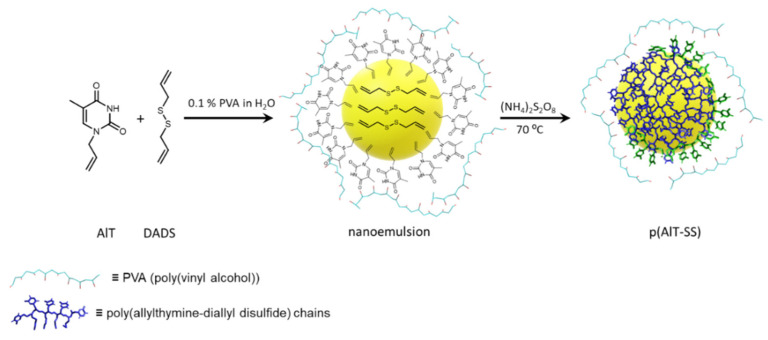
Synthesis of p(AlT-SS).

In this study, we propose a simple procedure for synthesizing a nanocarrier for DOX delivery. It consists in creating a nanoemulsion with its subsequent polymerization by stimulus-sensitive fragments. Diallyl disulfide (DADS) and 1-allylthymine (AlT) were employed as building components (Scheme 1). Being a lipophilic compound, DADS builds a dispersed phase, which then forms the redox-sensitive polymer core of the nanocarrier [27]. Thymine (5-methyluracil) functions as an emulsifier of the nanoemulsion, being soluble in both organic and aqueous media. Thymine and other uracil derivatives are frequently used as building blocks for the creation of medications with a variety of pharmacological activities [28,29,30]. Uracil derivatives demonstrate anti-inflammatory properties, promote cell development, boost immunity, and drive nucleic and protein metabolism [31]. They can also pass through the BBB into the brain tissues, inhibiting acetylcholinesterase [32]. Additionally, uracil conjugates with pharmacophores, such as DOX, and demonstrates promising outcomes in terms of cancer cell selectivity and targeted drug delivery [33,34,35]. Polyvinyl alcohol (PVA) was applied as an additional emulsifier of the nanoemulsion due to such characteristics as biodegradability, biocompatibility, and nontoxicity [36]. The article discusses the creation of the nanocarrier and its chemical and physical properties, such as size, molecular weight, and surface potential. The results about DOX ability to be transported by nanocarriers and released in an environment similar to that of cancer cells are provided. The data of biological studies, including the glioblastoma-cell-specific cytotoxicity of DOX nanocarrier composite, its glioblastoma cell penetration, and reactive oxygen species generation, are also discussed.

## 2. Results and Discussion

### 2.1. Synthesis and Characterization of p(AlT-SS) and DOX@p(AlT-SS)

A nanocarrier (p(AlT-SS), Scheme 1) was synthesized by nanoemulsion polymerization of AlT and DADS in the presence of PVA. DADS is a lipophilic compound that is insoluble in water, while AlT can dissolve in both organic and aqueous media. It organizes at the interface of the nanoemulsion, reducing the surface tension [37]. Allyl groups are directed to the dispersed phase—diallyl disulfide and thymine fragments—in the aqueous medium. 0.1% PVA was further added to the emulsion to stabilize it [38].

**Figure 1:**
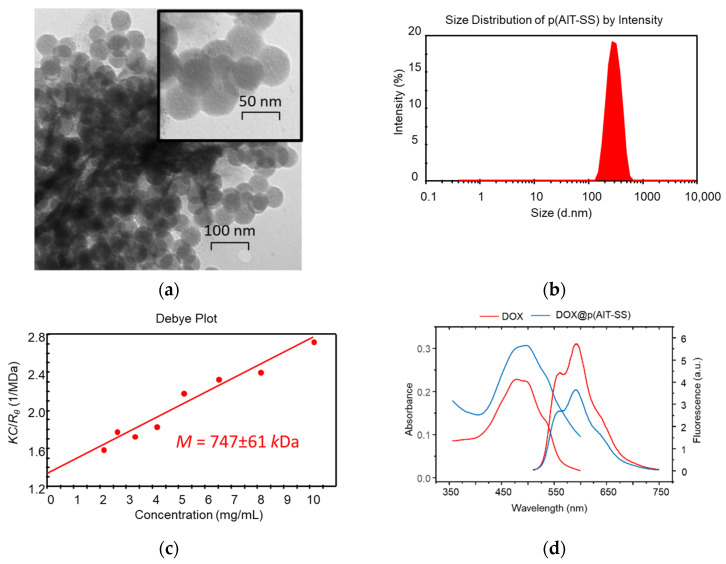
Data for p(AlT-SS): (**a**) TEM image; (**b**) Distribution diagram of the hydrodynamic diameter, 2 mg/mL, H_2_O, 25 °C; (**c**) Debye plot for the SLS analysis in the range 2–10 mg/mL, H_2_O, 25 °C; (**d**) UV–VIS and fluorescence spectra of DOX and DOX@p(AlT-SS), *C*(DOX) = 0.02 mM, H_2_O, 37 °C.

To obtain a nanoemulsion, 15 μL of DADS was added to a 10 mM AlT solution in 0.1% PVA aqueous solution. The mixture was homogenized on a vortex (3000 rpm) for 1 min and then kept in an ultrasonic bath for 1.5 h, purging with argon. The dispersed solution was heated to 70 °C, and 4% by weight of ammonium persulfate was added to promote the polymerization of DADS and the cross polymerization of AIT and DADS. Then the nanoemulsion was stirred for 14 h at a temperature of 70 °C. After that, the dispersed solution was dialyzed three times against water, and the solvent was removed under reduced pressure, resulting in the nanocarrier p(AlT-SS). The yield was 43.5% of the sum of AlT, DADS, and PVA. The transmission electron microscopy (TEM) image shows that p(AlT-SS) is approximately 50 nm in size (Figure 1a). According to dynamic light scattering (DLS) data, the hydrodynamic diameter is 328 ± 14 nm, which is substantially greater and indicates that the particles aggregate in water (Figure 1b). The low zeta potential (+0.1 mV) confirms the assembly tendency of p(AlT-SS). However, p(AlT-SS) is stable in water and has not precipitated or increased turbidity for several days. The Debye plot created from the static light scattering (SLS) data was used to calculate the molecular weight (*M*). The intercept of the Debye curve (*KC*/*R_θ_* vs. *C*, where *K* is the Debye constant, *C* is the concentration of p(AlT-SS), and *R_θ_* is the Rayleigh ratio) is equivalent to the *M* inverse and is around 0.00145 ± 0.0001 1/kDa, whereas *M* is 747 ± 61 kDa (Figure 1c).

**Figure 2:**
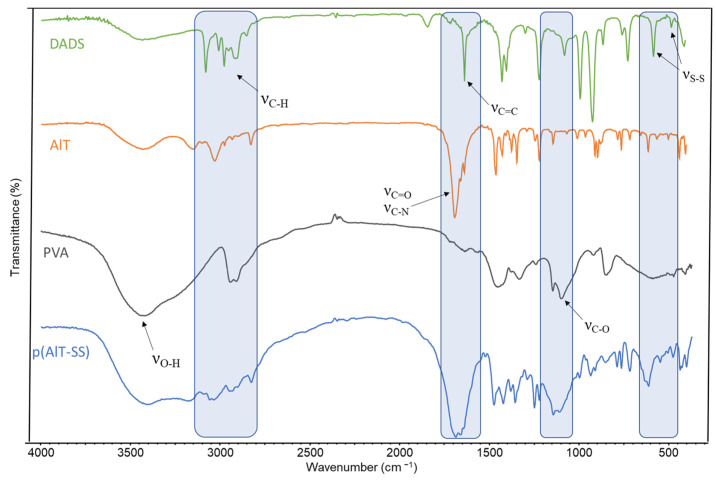
Comparison between the IR spectrum of p(AlT-SS) and DADS, AlT, and PVA.

The presence of AlT, DADS, and PVA fragments in the nanocarrier structure is confirmed by the IR spectrum, which contains characteristic vibration bands for all these compounds (Figure 2). A set of the C-H stretching vibration bands of AlT, DADS, and PVA is observed in the 2800–3200 cm^−1^ range. At 3400 cm^−1^, a broad band of the PVA O-H vibrations is visible. The PVA C-O bond vibrations are detected at 1100 and 1145 cm^−1^. The thymine C-N and C-O bonds vibrate at 1687 and 1661 cm^−1^. The S-S vibration signals of the nanocarrier core appear at 570 and 475 cm^−1^.

For DOX encapsulation and the formation of the composition drug/nanocarrier (DOX@p(AlT-SS)), 1.8 mg of DOX was added to the AlT/DADS/PVA nanoemulsion. The dispersed solution was vigorously stirred, and the polymerization procedure was followed exactly as for p(AlT-SS). At the end of the reaction, the solution was dialyzed from unencapsulated DOX. The amount of encapsulated DOX was calculated using the optical density of the DOX absorption band at 481 nm (*ε* = 10,410 M^−1^·cm^−1^). The encapsulation efficiency (*EE*%) was 18.6%, and the load capacity (*LC*%) was 4.6%.

The UV–VIS spectrum of DOX@p(AlT-SS) is similar to the UV–VIS spectrum of free DOX in the region of 350–600 nm, except for the increased baseline caused by light scattering when passing through the dispersed solution (Figure 1d). Because of the self-quenching processes [39] occurring inside the nanocarrier and screening of DOX by the nanocarrier shell, the fluorescence intensity of DOX@p(AlT-SS) is much lower than that of free DOX (Figure 1d).

Glutathione (GSH), the tripeptide glutamylcysteinylglycine, is found in almost all cells and performs a number of important cell functions. Malignant tumors frequently have higher GSH concentrations than healthy tissue. It enhances cell survival and shields them from anticancer drugs [40,41]. As a result, lowering the GSH level in the tumor can dramatically increase the efficacy of anticancer therapy.

**Figure 3:**
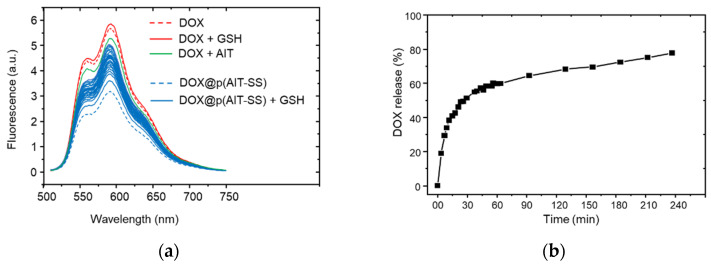
(**a**) Fluorescence spectra of DOX and its mixtures with AlT and GSH and temporal fluorescence spectra of DOX@p(AlT-SS) before and after adding GSH, *C*(DOX) = 0.02 mM, *C*(AlT) = 0.8 mM, *C*(GSH) = 8 mM, H_2_O, 37 °C; (**b**) DOX release after adding GSH to DOX@p(AlT-SS), *C*(DOX) = 0.02 mM, *C*(GSH) = 8 mM, H_2_O, 37 °C.

The core of DOX@p(AlT-SS) is composed of disulfide bonds produced by DADS. The disulfide bonds can interact with GSH to significantly reduce the quantity of GSH present in cancer cells. Additionally, the interaction between DOX@p(AlT-SS) and GSH leads to the dissociation of the nanocarrier and release of DOX. Indeed, fluorescence spectroscopy reveals that adding GSH to DOX@p(AlT-SS) increases fluorescence intensity, indicating DOX release from the nanocarrier (Figure 3a). A sharp release of doxorubicin occurs in the first hour (60%), and then another 20% release is observed within 3 h. At 4 h after the addition of GSH, almost 80% of DOX was released from the nanocarrier (Figure 3b). Free DOX, in contrast to DOX@p(AlT-SS), is mostly unaffected by GSH. With the addition of GSH, the fluorescence intensity of free DOX does not significantly change. AlT also has just a minor effect on the fluorescence of DOX (Figure 3a).

### 2.2. Biological Investigations

A line of healthy human liver cells (Chang liver) (Ch.L.) and lines of cancerous human cells (M-HeLa and glioblastoma T98G) were subjected to cytotoxic research. The results demonstrate that p(AlT-SS) is not toxic. The half-maximal cell growth inhibitory concentration (IC_50_) is far higher than the concentration required to provide the therapeutic dose of DOX (Table 1).

The DOX encapsulation in the nanocarrier leads to an enhanced cytotoxic effect of DOX on cancer cells. As a result, the IC_50_ of DOX@p(AlT-SS) for M-HeLa is 1.5 times lower than that of free DOX (Table 1). The greatest cytotoxic effect was observed on the glioblastoma cells T98G. Compared with free DOX, DOX@p(AlT-SS) has an almost threefold lower IC_50_. At the same time, the DOX cytotoxicity to healthy cells does not change significantly after encapsulation. The hypervascularization of tumor tissues, which results in increased tumor permeability and greater nanocarrier accumulation in the tumor compared with normal tissue, may be the reason for the selective impact on cancer cell lines [42]. The issue of selective action on glioblastoma cells is open. Glioblastoma differs significantly from other tumors. It exists only in the central nervous system, does not colonize elsewhere, and has a significantly distinct structure [43]. In the future, a more thorough investigation into this matter will be required.

**Figure 4:**
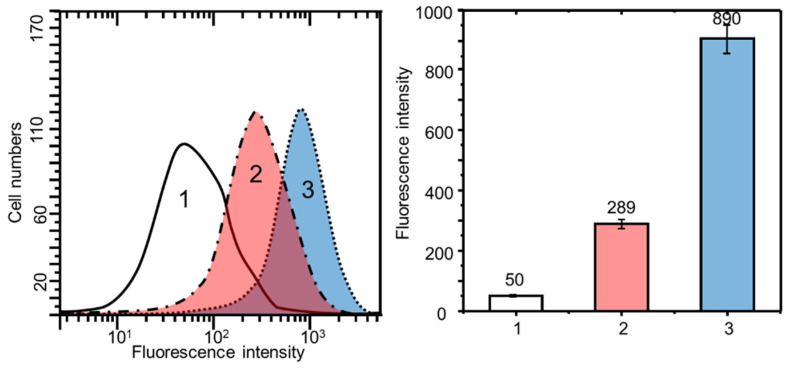
The log10 fluorescence intensity histogram and mean fluorescence intensity values: 1—control, 2—DOX, and 3—DOX@p(AlT-SS).

The penetration of DOX into T98G cells was evaluated using the fluorescent flow cytometry technique, which includes detecting the fluorescence of cells. DOX emits a bright red light. The amount of DOX that has entered the cells can be calculated by monitoring the fluorescence of the cells. Cells that were not treated served as a negative control. Data from flow cytometry revealed that p(AlT-SS) improved DOX penetration into cells (T98G). Incubation in DOX@p(AlT-SS) solution increases threefold the intensity of cell fluorescence compared with incubation in free DOX solution (Figure 4).

**Figure 5:**
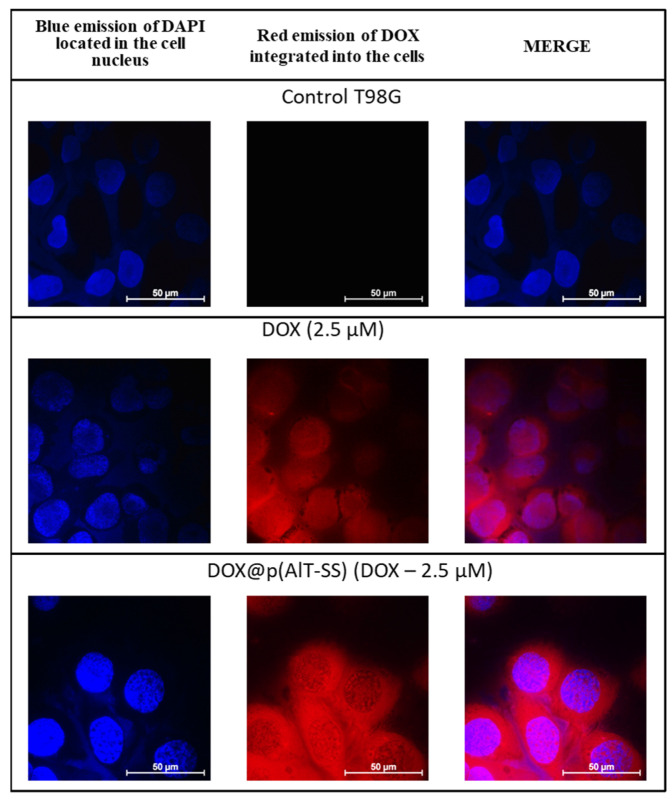
Images of a glioblastoma cell line (T98G) after incubation in DOX and DOX@p(AIT-SS) (Nikon Eclipse Ci, ×1000 oil), *C*(DOX) = 2.5 μM.

Fluorescence microscopy was used to assess the penetration of DOX@p(AlT-SS) into T98G cells. The DNA intercalating dye DAPI (4′,6-diamidino-2-phenylindole) was applied to label cell nuclei (blue emission). DOX shows red emission. The microscope images show that there are no discernible changes in cell morphology after the treatment with free DOX, which is mainly localized in the nucleus. Significant changes appear after the DOX@p(AlT-SS) action. The number of surviving cells is much lower. The nuclei of the cells are enlarged. The appearance of holes in the shell of the nucleus and the distribution of DOX throughout the entire volume of the cell are also observed (Figure 5).

**Figure 6:**
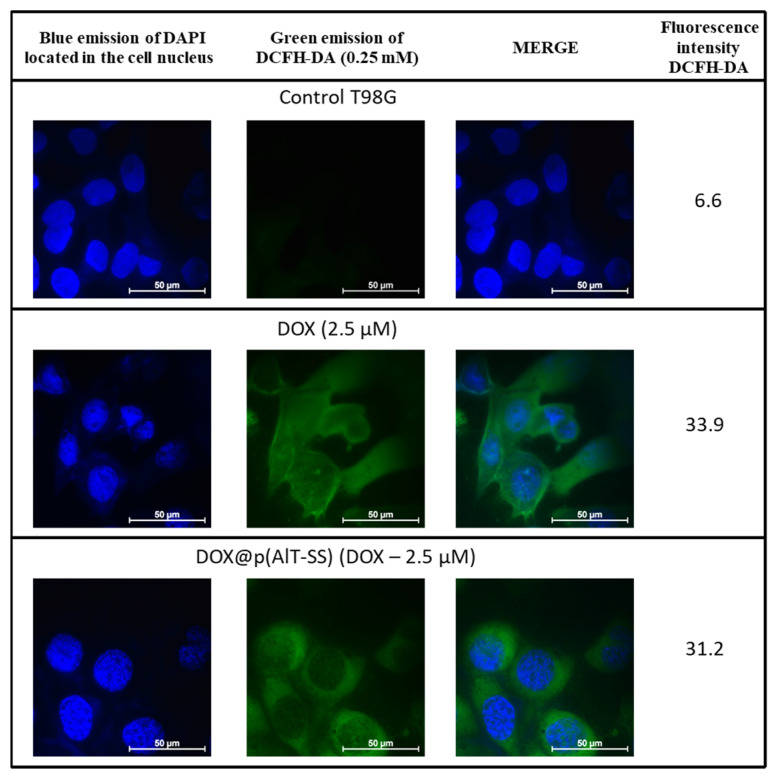
Production of ROS by DOX and DOX@p(AlT-SS), *C*(DOX) = 2.5 μM.

The action of DOX is based on the production of reactive oxygen species (ROS), which are involved in redox reactions and damage proteins and DNA [44]. Fluorescence microscopy was used to examine the formation of ROS in T98G cells in response to the DOX@p(AlT-SS) treatments (Figure 6). 2′,7′-Dichlorofluorescein diacetate (DCFH-DA) dye was used to determine ROS. It is evident that the ROS level increases significantly following treatment with DOX and DOX@p(AlT-SS). However, the quantity of ROS is slightly higher with free DOX. There are most likely fewer cells left after processing with DOX@p(AlT-SS).

## 3. Materials and Methods

Transmission electron microscopy (TEM) images were obtained using a Libra 120 EFTEM (A Carl Zeiss SMT AG Company, Oberkochen, Germany). The images were acquired at an accelerating voltage of 100 kV. The samples were dispersed on a 300 mesh copper grid with a continuous carbon/formvar support film. A Zetasizer Nano instrument (Malvern, UK) equipped with a 4 mW He–Ne solid-state laser operating at 633 nm was used for SLS and DLS experiments and zeta potential measurement. The Malvern Dispersion Technology software 5.0 was used for the analysis of particle size, molecular weight, and zeta potential. NMR spectra were recorded on a Bruker Avance 600 MHz spectrometer. IR spectra were recorded using a Vector-27 FTIR spectrometer (Bruker, Ettlingen, Germany) in the 400–4000 cm^−1^ range. The samples were prepared as KBr pellets. UV–Vis spectra were recorded with a PerkinElmer Lambda 25 UV–Vis spectrometer. A cuvette with an optical path length of 1 cm was used in all experiments. Fluorescence emission spectra were recorded with a Cary Eclipse fluorescence spectrophotometer (Agilent, Palo Alto, CA, USA). A quartz cell with a 1 cm path length was used for all fluorescence measurements. The excitation wavelength was 490 nm. A ViBRA AF-225 DRCE electronic analytical balance (220/0.001 g, resolution of 0.00001 g) was used to weigh the samples. Ultrasonic treatment was carried out on a “Sapphire” UZV 4.0 ultrasonic bath with a power of 280 W and an operating frequency of 35 kHz. A Multi Speed Vortex MSV-3500 Biosan was used for sample mixing.

AlT was prepared according to the method described in [45]. Commercially available DADS (80%, Sigma-Aldrich, St Louis, MO, USA), GSH (98%, Acros, Geel, Belgium), (NH_4_)_2_S_2_O_8_ (98%, Acros, Geel, Belgium), PVA (88%, Acros, Geel, Belgium, *M* = 88,000), DAPI (98%, Sigma-Aldrich, St Louis, MO, USA), and DCFH-DA (95%, Sigma-Aldrich, St Louis, MO, USA) were used without purification. The water was purified using an Adrona Crystal E purification system.

### 3.1. Synthesis of p(AlT-SS)

In 1 mL of 0.1% PVA aqueous solution, AlT (1.7 mg, 10 μmol) was dissolved. An amount of 15 μL of DADS was added, and the mixture was homogenized first on a vortex at 3500 rpm for 1 min, and then in an ultrasonic bath for 90 min with simultaneous purging with argon. The resulting dispersed solution was heated with stirring at 70 °C for 30 min, and then 0.67 mg of (NH_4_)_2_S_2_O_8_ in 100 μL of water (4% of the mass sum of AlT, DADS) was added. The mixture was heated for 14 h by stirring. After that, the solution was dialyzed three times for 60 min using a 2000 Da dialysis bag. The precipitate was dried at room temperature after the solvent was removed, yielding p(AlT-SS) as a white solid. Yield: 7.25 mg (43.5%). IR (KBr, cm^−1^, ν_max_): 3400 (O-H); 3180, 3035, 2947, 2827 (C–H); 1687 (C=O); 1661 (C–N); 1145, 1100 (C–O); 570, 475 (S–S). Anal. Calcd. for ((C_8_H_10_N_2_O_2_)_3_·C_6_H_10_S_2_·(C_2_H_4_O)_5_·(H_2_O)_6_)*_n_*, %: C 49.37; H 7.46; N 8.64; O 27.95; S 6.59. Found C 48.88; H 6.97; N 8.98; S 6.18.

### 3.2. Encapsulation of DOX in p(AlT-SS) (DOX@p(AlT-SS))

A solution of DOX (1.88 mg) and AlT (1.7 mg, 10 mol) in 0.1% PVA aqueous solution (1 mL) was treated with 15 μL of DADS. The mixture was vortexed for 1 min (3500 rpm). The resulting suspension was bubbled with argon and sonicated for 90 min. The resulting solution was heated to 70 °C, and 0.67 mg of (NH_4_)_2_S_2_O_8_ in 0.1 mL of water was added. The mixture was stirred for 14 h at 70 °C. Then the solution was dialyzed three times for 60 min using a 2000 Da dialysis bag.

The DOX@p(AlT-SS) solution was diluted 10 times, and the amount of encapsulated DOX was calculated using the formula:

$$m_{DOX}=\left(A_{481}-A_{481base}\right)\times 10\times M/\varepsilon_{481},$$

where *m_DOX_* is the mass of the encapsulated DOX, in mg; *A*_481_ is the optical density of the solution DOX@p(AlT-SS) at 481 nm; *A*_481base_ is the optical density of the baseline at 481 nm; *M* is the molecular weight of DOX, in g/mol; and ε_481_ is the molar extinction coefficient of DOX at 481 nm, which is equal to 10,410 M^−1^·cm^−1^.

*EE*% was determined as the ratio of the mass of encapsulated DOX to the initially taken amount:

$$EE\%=m_{DOX}/m_{DOX}^{0}\times 100\%\;$$

where *m_DOX_* is the mass of the encapsulated DOX, in mg, and $m_{DOX}^{0}$ is the mass of DOX that was taken for the reaction, in mg.

*LC*% was determined by the formula:

$$LC\%=m_{DOX}/(m_{AlT}+m_{DADS})\times 100\%,$$

where *m_DOX_* is the mass of the encapsulated DOX, in mg, and *m_AlT_* and *m_DADS_* are the masses of AlT and DADS, respectively, used in the synthesis of DOX@p(AlT-SS).

The DOX@p(AlT-SS) solution obtained was diluted 30.7 times for fluorescent and biological experiments, yielding a DOX concentration of 0.02 mM.

### 3.3. Cytotoxicity Assay

The cytotoxic effect of glutathione responsive nanocarrier on cancerous and normal human cells was assessed by a fluorescent method using the Cytell multifunctional cell imaging system (GE Healthcare Life Science, Uppsala, Sweden) using the Cell Viability Bio App. Two fluorescent dyes were used in the experiments (DAPI and propidium iodide, which selectively penetrate cell membranes and fluoresce at different wavelengths). As a result, living cells were stained blue, and dead cells were stained orange [46]. M-HeLa clone 11 humans, epithelioid cervical carcinoma, the strain of HeLa, the clone of M-HeLa, glioblastoma cell line (T98G) from the Collection of Type Cultures of the Institute of Cytology of the Russian Academy of Sciences, and the Chang liver cell line (human liver cells) from N. F. Gamaleya National Research Center for Epidemiology and Microbiology were used in the experiments. The cells were grown on a standard nutrient medium Eagle produced by the Research Institute of Poliomyelitis and Viral Encephalitis, Chumakov (PanEco Company, Moscow, Russia), with the addition of 10% fetal calf serum and 1% nonessential amino acids. Cells were seeded in a 96-well plate at a concentration of 1 × 10^5^ cells/mL. An amount of 150 μL of cell suspension was added to each well and cultured in a CO_2_ incubator at 37 °C. Twenty-four hours after seeding the cells in the wells, the test compound was added at a predetermined dilution of 150 μL to each well. Compound dilutions were prepared directly on nutrient media. The experiments were repeated three times. Intact cells cultured in parallel with the experimental cells were used as controls.

### 3.4. Flow Cytometry Assay

#### 3.4.1. Cell Culture

T98G cells in the amount of 1 × 10^5^ cells/well at a final volume of 2 mL were seeded in 6-well plates. After 24 h incubation, DOX and DOX@p(AlT-SS) were added to the wells and cultivated for 24 h in a CO_2_ incubator. Cellular uptake was analyzed by flow cytometry (Guava easyCyte 8HT, Luminex, Austin, TX, USA). Untreated cells were used as a negative control. The experiments were repeated three times.

#### 3.4.2. Fluorescence Microscopy

T98G cells at an amount of 1 × 10^5^ cells/well at a final volume of 2 mL were seeded in 6-well plates with coverslips at the bottom of each well. After 24 h incubation, DOX and DOX@p(AlT-SS) were added to the wells and cultivated for 24 h in a CO_2_ incubator. Then T98G cells were fixed and stained with DAPI (blue). Studies were performed using a Nikon Eclipse Ci-S fluorescence microscope (Nikon, Tokyo, Japan) at a magnification of 1000×.

### 3.5. Induction of the Production of Intracellular ROS

#### Fluorescence Microscopy

To detect ROS, the treatment T98G cells were harvested at 2000 rpm for 5 min and then washed twice with ice-cold phosphate-buffered saline (PBS), followed by resuspension in 0.5 mL growth media without PBS containing 5 μM of DCFH-DA and incubated at 37 °C for 1 h. After washing the cells three times in PBS, the ROS production in the cells was immediately monitored using a Nikon Eclipse Ci-S fluorescence microscope (Nikon, Tokyo, Japan) at a magnification of 1000×. The fluorescence intensity (DCFH-DA) was determined using NISE software elements, designed for the analysis and processing of information obtained from a Nikon Eclipse Ci-S microscope.

### 3.6. Statistical Analysis

IC_50_ was calculated using an online tool: MLA—“Quest Graph™ IC50 Calculator” (AAT Bioquest, Inc., https://www.aatbio.com/tools/ic50-calculator (accessed on 5 July 2021)) [47].

The cytometric results were analyzed by the Cytell Cell Imaging multifunctional system using the Cell Viability BioApp. The dat a in the tables and graphs are given as the mean ± standard error.

## 4. Conclusions

A new nanocarrier was produced by nanoemulsion polymerization of allylthymine and diallyl disulfide for the delivery of anticancer drugs. Unlike previously known carriers, the nanocarrier is easily obtainable, stable, and nontoxic. The nanocarrier facilitates the drugs’ penetration into cancer cells, as shown in the drug doxorubicin. Microbiological studies have shown that doxorubicin encapsulated in the nanocarrier exhibits specific cytotoxicity to T98G glioblastoma cancer cells in contrast to a healthy cell line (Ch.L.) and a cancer cell line (M-HeLa).

## Figures and Tables

**Table 1:** IC_50_ for p(AIT-SS), DOX, and DOX@p(AIT-SS) obtained from the cancer cell line M-HeLa glioblastoma and for the healthy cell line Ch.L.

Compounds	IC_50_ (mg/mL)		
	Cancer Cells		Healthy Cells
	M-HeLa	T98G	Ch.L.
p(AlT-SS)		>0.2	
DOX	0.0032 (0.006 mM) ^1^	0.0032 (0.006 mM) ^1^	0.0016 (0.003 mM) ^1^
DOX@p(AlT-SS)	0.024 (0.002 mM) ^1^	0.008 (0.0007 mM) ^1^	0.035 (0.003 mM) ^1^

^1^ DOX concentration.

## Data Availability

Not applicable.
